# Ferroptosis: past, present and future

**DOI:** 10.1038/s41419-020-2298-2

**Published:** 2020-02-03

**Authors:** Jie Li, Feng Cao, He-liang Yin, Zi-jian Huang, Zhi-tao Lin, Ning Mao, Bei Sun, Gang Wang

**Affiliations:** 1 Department of Pancreatic and Biliary Surgery, The First Affiliated Hospital of Harbin Medical University, Harbin, Heilongjiang Province China; 2 Key Laboratory of Hepatosplenic Surgery, Ministry of Education, The First Affiliated Hospital of Harbin Medical University, Harbin, China; 3 General Surgery Department, Xuanwu Hospital, Capital Medical University, Beijing, China; 4 Department of General Surgery, The First Hospital of Qiqihar, Qiqihar, Heilongjiang 161005 China; 5 Department of General Surgery, Affiliated Qiqihar Hospital, Southern Medical University, Qiqihar, Heilongjiang 161007 China

**Keywords:** Cell death, Cell signalling

## Abstract

Ferroptosis is a new type of cell death that was discovered in recent years and is usually accompanied by a large amount of iron accumulation and lipid peroxidation during the cell death process; the occurrence of ferroptosis is iron-dependent. Ferroptosis-inducing factors can directly or indirectly affect glutathione peroxidase through different pathways, resulting in a decrease in antioxidant capacity and accumulation of lipid reactive oxygen species (ROS) in cells, ultimately leading to oxidative cell death. Recent studies have shown that ferroptosis is closely related to the pathophysiological processes of many diseases, such as tumors, nervous system diseases, ischemia-reperfusion injury, kidney injury, and blood diseases. How to intervene in the occurrence and development of related diseases by regulating cell ferroptosis has become a hotspot and focus of etiological research and treatment, but the functional changes and specific molecular mechanisms of ferroptosis still need to be further explored. This paper systematically summarizes the latest progress in ferroptosis research, with a focus on providing references for further understanding of its pathogenesis and for proposing new targets for the treatment of related diseases.

## Facts

- Ferroptosis is a new type of programmed cell death, which occurs with iron dependence.
- Ferroptosis plays an important regulatory role in the occurrence and development of many diseases, such as tumors, neurological diseases, acute kidney injury, ischemia/reperfusion, etc.
- Activating or blocking the ferroptosis pathway to alleviate the progression of the disease, which provides a promising therapeutic strategy for many diseases.

## Open questions

- What is the relationship between ferroptosis and other types of cell death? Is it synergy or antagonism?
- Is iron necessary to promote the production of lipid peroxides, or can other substances take the place of iron in ferroptosis?
- What is the downstream regulation mechanism of iron in ferroptosis?
- How can ferroptosis promote the development of inflammation?

## Introduction

**Fig. 1:**
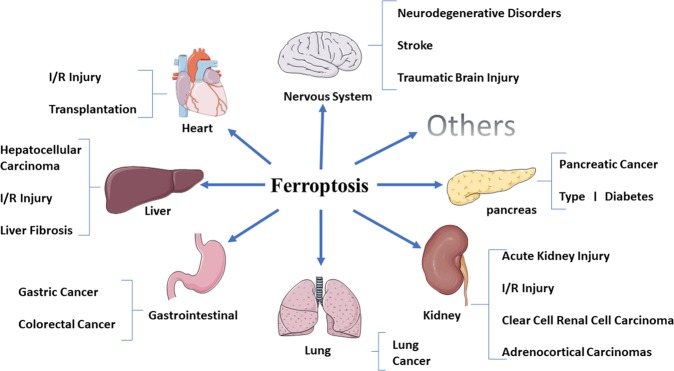
Ferroptosis has played important roles in multiple system diseases, such as nervous system diseases, heart diseases, liver diseases, gastrointestinal diseases, lung diseases, kidney diseases, pancreatic diseases, and so on.

Whether under physiological or pathological conditions, cell death is an unavoidable and important link in the process of life and marks the end of the life of a cell. Traditionally, cell death has been divided into apoptosis and necrosis. Recent studies have shown that in addition to necrosis and apoptosis, there are also other new programmed death modes, such as autophagy, necrosis and necrotic apoptosis, which have unique biological processes and pathophysiological characteristics. In 2012, Dixon^1^ first proposed the concept of ferroptosis, an iron-dependent, non-apoptotic mode of cell death characterized by the accumulation of lipid reactive oxygen species (ROS). Ferroptosis is obviously different from necrosis, apoptosis, and autophagy in cell morphology and function (Table 1)^1,2^. It does not have the morphological characteristics of typical necrosis, such as swelling of the cytoplasm and organelles and rupture of the cell membrane, nor does it have the characteristics of traditional cell apoptosis, such as cell shrinkage, chromatin condensation, formation of apoptotic bodies and disintegration of the cytoskeleton. In contrast to autophagy, ferroptosis does not have the formation of classical closed bilayer membrane structures (autophagic vacuoles). Morphologically, ferroptosis mainly manifests as obvious shrinkage of mitochondria with increased membrane density and reduction in or vanishing of mitochondrial cristae, which is a different process from other modes of cell death^1,3,4^. Recent studies have shown that ferroptosis plays an important regulatory role in the occurrence and development of many diseases and has become the focus and hotspot of research on the treatment and prognosis improvement of related diseases (Fig. 1). In view of this, we reviewed the latest research progress on ferroptosis to provide some references for further understanding of its pathogenesis and for proposing new targets for the treatment of related diseases.

**Table 1:** The features of ferroptosis, apoptosis, autophagy, and necroptosis

	Ferroptosis	Apoptosis	Autophagy	Necroptosis
Morphological Features	Small mitochondria with increased mitochondrial membrane densities, reduction or vanishing of mitochondria Crista, outer mitochondrial membrane Rupture and normal nucleus	Cellular and nuclear volume reduction, chromatin agglutination, nuclear fragmentation, formation of apoptotic bodies and cytoskeletal disintegration, no significant changes in mitochondrial structure	Formation of double-membraned autolysosomes, including macroautophagy, microautophagy and chaperone-mediated autophagy	Plasma membrane breakdown, generalized swelling of the cytoplasm and organelles, moderate chromatin condensation, spillage of cellular constituents into the microenvironment
Biochemical Features	Iron accumulation and lipid peroxidation	DNA fragmentation	Increased lysosomal activity	Drop in ATP levels
Regulatory Pathways	Xc- /GPX4, MVA, sulfur transfer pathway, P62-Keap1-NRF2 pathway, P53/SLC7A11, ATG5-ATG7-NCOA4 pathway, P53-SAT1-ALOX15 pathway, HSPB1-TRF1, FSP1-COQ10-NAD(P)H pathway	Death receptor pathway, mitochondrion pathway and endoplasmic reticulum pathway; Caspase, P53, Bcl-2 mediated signaling pathway	mTOR, Beclin-1, P53 signaling pathway	TNF-R1 and RIP1/RIP3-MLKL related signaling pathways; PKC-MAPK-AP-1 related signaling pathway; ROS-related metabolic regulation pathway
Key genes	GPX4, TFR1, SLC7A11, NRF2, NCOA4, P53, HSPB1, ACSL4, FSP1	Caspase, Bcl-2, Bax, P53, Fas	ATG5, ATG7, LC3, Beclin-1, DRAM3, TFEB	RIP1, RIP3

*ACSL4* acyl-CoA synthetase long-chain family member 4, *ALOX-15* arachidonate lipoxygenase 15, *AP-1* activator protein-1, *ATG5* autophagy-related 5, *ATG7* autophagy-related 7, *COQ10* coenzyme Q10, *DRAM3* damage-regulated autophagy modulator 3, *FSP1* ferroptosis suppressor protein 1, *GPX4* glutathione peroxidase 4, *HSPB1* heat shock protein beta-1, *Keap1* Keleh-like ECH-associated protein 1, *MAPK* mitogen-activated protein kinase, *MLKL* mixed lineage kinase domain like protein, *mTOR* mammalian target of rapamycin, *MVA* mevalonate, *LC3* microtubule-associated protein 1 light chain3, *NCOA4* nuclear receptor coactivator 4, *NRF2* nuclear factor erythroid 2-related factor 2, *PKC* protein kinase C, *RIP* receptor-interacting serine/threonine kinase, *ROS* reactive oxygen species, *SAT1* spermidine/spermine N1-acetyltransferase 1, *SLC7A11* solute carrier family 7 member 11, *system Xc-* cysteine/glutamate transporter receptor, *TFEB* transcription factor EB, *TFR1* transferrin receptor 1, *TNF-R1* tumor necrosis factor R1.

## An overview of ferroptosis

In 2003, Dolma^5^ et al. discovered a new compound, erastin, which had a selectively lethal effect on RAS-expressing cancer cells, but the manner of cell death was different from what had been seen before. There were no nuclear morphological changes, DNA fragmentation, and caspase activation, and this process could not be reversed by caspase inhibitors. Subsequently, Yang^3^ and Yagoda^4^ found that this cell death pattern could be inhibited by iron chelating agents and found another compound, RSL3, which could cause this pattern of cell death. In 2012, Dixon^1^ et al. formally named this cell death ferroptosis, according to its characteristics when studying the mechanism by which erastin killed cancer cells with RAS mutations. Ferroptosis is a new mode of cell death. Morphologically, ferroptosis occurs mainly in cells as reduced mitochondrial volume, increased bilayer membrane density and reduction or disappearance of mitochondrial cristae^1,3^, but the cell membrane remains intact, the nucleus is normal in size, and there is no concentration of chromatin; biochemically, there is intracellular glutathione (GSH) depletion and decreased activity of glutathione peroxidase 4 (GPX4), lipid peroxides cannot be metabolized by the GPX4-catalyzed reduction reaction, and Fe^2+^ oxidizes lipids in a Fenton-like manner, resulting in a large amount of ROS, which promotes ferroptosis^3,6^; and genetically, ferroptosis is a biological process regulated by multiple genes. Ferroptosis mainly involves genetic changes in iron homeostasis and lipid peroxidation metabolism, but the specific regulatory mechanism needs to be further studied. A variety of substances that induce ferroptosis can be divided into four categories. One category includes erastin, which is the prototype ferroptosis inducer that reduces GSH levels by directly inhibiting system Xc-. Erastin, however, also has another target, voltage-dependent anion channels (VDACs), which induces mitochondrial dysfunction. Recently, it was also found that activation of ferroptosis by erastin increases the level of lysosomal-associated membrane protein 2a, thereby promoting chaperone-mediated autophagy, which in turn promotes the degradation of GPX4^7^. The second category includes RSL3 and DPI7, which directly inhibit GPX4 activity induce ferroptosis. The third category includes FIN56, which has two methods of inducing ferroptosis. First, FIN56 promotes GPX4 degradation. Second, FIN56 binds to the enzyme squalene synthase, which leads to the depletion of endogenous antioxidant coenzyme Q10 (COQ10). This process enhances cell sensitivity to FIN56-induced ferroptosis^8^. The final category includes FINO2, an organic peroxide with many features in common with artemisinin, which causes ferroptosis due to a combined effect of the direct oxidation of labile iron and the inactivation of GPX4^8^. With the deepening of research on the mechanism of ferroptosis, many specific inhibitors of ferroptosis, such as ferrostatin-1 (Fer-1), liproxstatin-1 and vitamin E, have been found, in addition to iron chelators. These substances inhibit ferroptosis by inhibiting the formation of lipid peroxides (Table 2). In 2014, Skouta et al. found that Fer-1 inhibited cell death in three in vitro models of Huntington’s disease (HD), periventricular white matter (PVL), and renal insufficiency. This provides the basis for the use of ferrostatin in disease models, and it is the first to address the importance of ferroptosis beyond the cell culture level^9^. In short, as a new manner of cell death, the discovery of ferroptosis provides a new way of thinking about and treating many diseases.

**Table 2:** The common inducers and inhibitors of ferroptosis.

	Mechanisms	Drugs or compounds
Inducer	Class1: Inhibit system Xc- and prevent cystine import	erastin, Sorafenib, Sulfasalazine
	Class2: Inhibit GPX4	RSL3, (1S,3R)-RSL3, DPI7, DPI10
	Class3: Degrade GPX4, bind to SQS and deplete antioxidant CoQ10	FIN56
	Class4: Oxidize ferrous iron and lipidome directly, inactivate GPX4 indirectly	FINO2
	Supplement: Target VDACs, degrade GPX4	erastin
Inhibitor	Class1: Inhibit accumulation of iron	DFO, Deferoxamine mesylate, 2,2’-pyridine
	Class2: Inhibit lipid peroxidation	Fer-1, SRS11–9, SRS16–86, Liproxststatin-1, Vitamin E

*Ace* acetaminophen, *ART* artesunate, *COQ10* coenzyme Q10, *DFO* deferoxamine, *Fer-1* Ferrostatin-1, *GPX4* glutathione peroxidase 4, *GSH* glutathione; *RSL3* Ras-selective lethal small molecule 3, *VDACs* voltage-dependent anion channels.

## Mechanism

### Inducing ferroptosis by suppressing system Xc-

System Xc- is an amino acid antitransporter that is widely distributed in phospholipid bilayers. It is part of an important antioxidant system in cells and is a heterodimer composed of two subunits, SLC7A11 and SLC3A2. Cystine and glutamate are exchanged in and out of the cell by system Xc- at a ratio of 1:1^1^. The cysteine that is taken up is reduced to cysteine in cells, which is involved in the synthesis of GSH. GSH reduces ROS and reactive nitrogen under the action of glutathione peroxidases (GPXs). Inhibiting the activity of system Xc- affects the synthesis of GSH by inhibiting the absorption of cystine, which leads to a decrease in GPX activity, a decrease in cell antioxidant capacity, accumulation of lipid ROS, and ultimately the occurrence of oxidative damage and ferroptosis. In addition, P53 can also inhibit system Xc- uptake of cystine by downregulating the expression of SLC7A11, thereby affecting the activity of GPX4, resulting in a reduction in cell antioxidant capacity, accumulation of lipid ROS, and ferroptosis^10,11^.

### Inducing ferroptosis by suppressing GPX4

Among the many members of the GPX family, GPX4 plays a pivotal role in the occurrence of ferroptosis and is the key regulator of its occurrence, mainly by inhibiting the formation of lipid peroxides. GPX4 converts GSH into oxidized glutathione (GSSG) and reduces the cytotoxic lipid peroxides (L-OOH) to the corresponding alcohols (L-OH). Inhibition of GPX4 activity can lead to the accumulation of lipid peroxides, which is a marker of ferroptosis. Yang et al.^3^ found that cells with downregulated GPX4 expression were more sensitive to ferroptosis, while the upregulation of GPX4 expression inhibits ferroptosis. RSL3, a ferroptosis inducer, directly acts on GPX4 and inhibits its activity, thus reducing the antioxidant capacity of cells and accumulating ROS, leading to ferroptosis^12^. In addition, the compounds DPI7 and DPI10 also directly act on GPX4 and induce ferroptosis. Selenocysteine is one of the essential amino acids of the GPX4 active group^12^. Selenocysteine tRNA is needed to insert selenocysteine into GPX4^13^. The mevalonate (MVA) pathway can affect the synthesis of GPX4 by regulating the maturation of selenocysteine tRNA, thereby regulating the occurrence of ferroptosis. In MVA pathway, IPP and COQ10 are two important products of this pathway^14^. Therefore, inhibiting the MVA pathway can downregulate the synthesis of selenocysteine tRNA, thereby affecting GPX4 activity and inducing ferroptosis.

### Ferroptosis mediated by mitochondrial VDACs

VDACs are transmembrane channels that transport ions and metabolites and play an important regulatory role in ferroptosis^15^. Yagoda et al.^4^ found that erastin acts on VDACs, leading to mitochondrial dysfunction and resulting in a large amount of released oxides, eventually leading to iron-mediated cell death.

### P53-Mediated ferroptosis

The P53 gene is an important tumor suppressor gene. P53-mediated cell cycle inhibition, aging, and apoptosis play important roles in the occurrence and development of tumors, but the role of P53 in ferroptosis is still unclear. Recently, acetylation-deficient P53 mutants have been found to promote ferroptosis. Jiang^10^ et al. found that the activity of H1299 cells with silenced P53 genes remained unchanged when treated with ROS. However, 90% of the cells died when treated with ROS after P53 activation, suggesting that activation of P53 reduces the antioxidant capacity of these cells. After treatment with Fer-1, a ferroptosis inhibitor, the cell death rate decreased significantly, and P53 also induced ferroptosis. Further studies have found that P53 can inhibit system Xc- uptake of cystine by downregulating the expression of SLC7A11, thereby affecting the activity of GPX4 and resulting in the reduction of antioxidant capacity, ROS accumulation, and ferroptosis^11^. In addition, the P53-SAT1-ALOX15 pathway is also involved in the regulation of ferroptosis^16^. SAT1 is a transcriptional target of P53 and an important rate-limiting enzyme for polyamine catabolism. Activation of SAT1 induces lipid peroxidation and ferroptosis induced by ROS, which is closely related to the expression level of arachidonate lipoxygenase 15 (ALOX-15). Other studies have shown that the expression of P53 also inhibits ferroptosis in some cells. Tarangelo A et al.^17^ found that stable wild-type P53 decreased the activity of system Xc- but also reduced the sensitivity of some cells to ferroptosis. Human HT-1080 fibroblasts are typical wild-type P53-expressing cells. When treated with the P53 inducer nutlin3, Amy and colleagues found that these cells were not sensitive to erastin-2-induced ferroptosis. Further studies showed that nutlin-3 increases the P53 level in wild-type U-2OS, ACHN, Caki-1 and A549 cells, which could resist ferroptosis induced by erastin-2. In addition, this process of reducing ferroptosis sensitivity requires the involvement of CDKN1A (encoding P21), a P53 transcription target that regulates GSH metabolism and intracellular GSH. These results indicate that the P53-P21 axis can negatively regulate the occurrence of ferroptosis in cancer cells. In addition, Xie et al.^18^ found that the expression of P53 was involved in the inhibition of ferroptosis in colorectal cancer cells. Therefore, P53 may regulate ferroptosis through a two-way pathway, but the specific mechanism still needs further study.

### Role of ferroptosis suppressor protein 1 (FSP1) (previously known as apoptosis-inducing factor mitochondrial 2 (AIF-M2))

Two recent studies have reported the role of FSP1/AIFM2 in the occurrence of ferroptosis, revealing a new effective way to regulate ferroptosis and reintroducing apoptosis-inducing factor (AIF)^19,20^. AIF was proposed before the concept of ferroptosis, and it has been involved in the study of apoptosis. Susin et al.^21^ identified AIF as a mitochondrial flavoprotein and a mitochondrial effector of apoptotic cell death while looking for molecules that induce noncaspase-dependent apoptosis. Recombinant AIF causes chromatin condensation in isolated nuclei and large-scale fragmentation of DNA. It induces purified mitochondria to release the apoptogenic proteins cytochrome c and caspase-9. As a flavoprotein, AIF-M2 is thought to be a p53-responsive gene and induces apoptosis by sequence similarity to another initially postulated proapoptotic gene, apoptosis-inducing factor mitochondria-associated 1 (AIFM-1)^22^. In a recent study, Bersuker et al.^19^ used a synthetic lethal CRISPR-Cas9 screen to confirmed that FSP1, formerly known as AIFM2, was an effective ferroptosis-resistance factor. Furthermore, Doll et al.^20^ also performed relevant research on FSP1. An expression cloning method was used to identify genes in human cancer cells that complement the loss of GPX4. It was then found that the flavoprotein AIFM2 was a previously unrecognized antiferroptotic gene, namely, FSP1, which had a protective effect on GPX4 deletion-induced ferroptosis. FSP1 knockout cell lines were significantly more sensitive to ferroptosis inducers and were rescued by overexpression of FSP1. In addition, the overexpression of FSP1 did not induce apoptosis, and activation of p53 did not increase FSP1 expression. Further studies have shown that the myristoylation of FSP1 mediates the recruitment of this protein to the plasma membrane, where it performs its function as an oxidoreductase that reduces COQ10 (also known as ubiquinone-10) and as a lipophilic radical-trapping antioxidant that halts the propagation of lipid peroxides. Doll et al.^20^ showed that FSP1 catalyzes the regeneration of COQ10 by NAD(P)H, and the FSP1-COQ10-NAD(P)H pathway is an independent parallel system that cooperates with GPX4 and glutathione to suppress phospholipid peroxidation and ferroptosis. Moreover, this also explains the effect of NAD(P)H in the MVA pathway through the loss of ubiquinone convergence on FSP1 and thereby predicts sensitivity to ferroptosis. In addition, the expression of FSP1 was positively correlated with ferroptosis resistance in hundreds of tumor cell lines, and FSP1 mediates ferroptosis resistance in lung cancer cells in culture and in mouse tumor xenografts. In conclusion, this study suggests that the discovery of FSP1 completes the ferroptosis pathway. The expression of FSP1 is critical for predicting the efficacy of ferroptosis-inducing drugs in cancers and has also identified the potential of FSP1 inhibitors as strategies to overcome ferroptosis resistance in many cancers.

### Role of iron metabolism in ferroptosis

Iron is an important trace element in the body. Abnormal distribution and content of iron in the body can affect the normal physiological processes. Fe^2+^ formed by intestinal absorption or erythrocyte degradation can be oxidized by ceruloplasmin to Fe^3+^, which binds to transferrin (TF) on the cell membrane to form TF-Fe^3+^, which forms a complex through membrane protein TF receptor 1 (TFR1) to endocytose this complex^23^. Fe^3+^ is then reduced to Fe^2+^ by six-transmembrane epithelial antigen of the prostate 3 (STEAP3), and Fe^2+^ is then stored in the unstable iron pool (LIP) and ferritin, which is mediated by divalent metal transporter 1 (DMT1) or Zinc-Iron regulatory protein family 8/14 (ZIP8/14). Excess Fe^2+^ is oxidized to Fe^3+^ by ferroportin (FPN)^24^. This recycling of internal iron strictly controls iron homeostasis in cells. Silencing TFRC, the gene encoding TFR 1, can inhibit erastin-induced ferroptosis^25^, while heme oxygenase-1 (HO-1) can accelerate erastin-induced ferroptosis by supplementing iron^26^. It was found that heat shock protein beta-1 (HSPB1) can further reduce intracellular iron concentrations by inhibiting TRF1 expression, and so overexpressed HSPB1 can significantly inhibit ferroptosis^27^. In addition, ferritin is composed of ferritin light chain (FTL) and ferritin heavy chain 1 (FTH1). Inhibiting the expression of iron response element binding protein 2 (IREB2), the main transcription factor of iron metabolism, can significantly increase the expression of FTL and FTH1, thereby inhibiting ferroptosis induced by erastin^28^.

### Regulation of the lipid metabolism pathway

Iron-dependent lipid ROS accumulation is involved in ferroptosis in all pathways. Lipid metabolism is closely related to ferroptosis. Polyunsaturated fatty acids (PUFAs) are sensitive to lipid peroxidation and are one of the essential elements for ferroptosis^29^. Free PUFAs are the substrate of the synthetic lipid signal transduction medium, but they must be esterified into membrane phospholipids and oxidized to transmit the ferroptosis signal. Studies have shown that phosphatidylethanolamine (PE), which contains arachidonic acid (AA) or its derivative adrenaline, is the key phospholipid that induces ferroptosis in cells. Acyl-CoA synthetase long-chain family member 4 (ACSL4) and lysophosphatidylcholine acyltransferase 3 (LPCAT3) participate in the biosynthesis and remodeling of PE, activate PUFAs and affect the transmembrane characteristics of PUFAs. Therefore, reducing the expression of ACSL4 and LPCAT3 reduces the accumulation of lipid peroxide substrates in cells, thus inhibiting ferroptosis. Ultimately, PUFA-PE can play further oxidative roles under the catalysis of lipoxygenase (LOX) and eventually induce ferroptosis in cells^30^.

### Other pathways

**Fig. 2:**
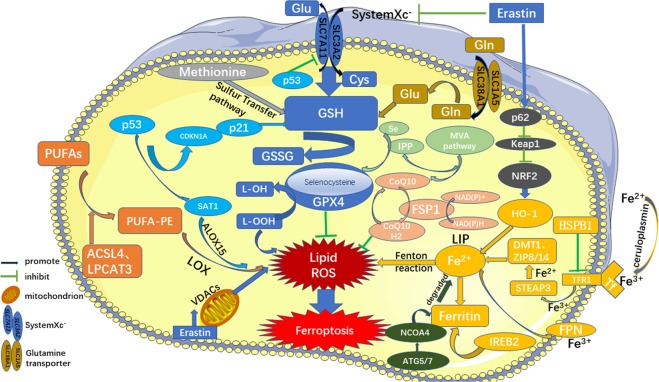
Regulatory pathways of ferroptosis. The figure shows the regulatory pathways of ferroptosis, which can be roughly divided into three categories. The first one is regulated by GSH/GPX4 pathway, such as inhibition of system Xc-, sulfur transfer pathway, MVA pathway, glutamine pathway, and p53 regulatory axis. Second, the regulation mechanism of iron metabolism, such as the regulation of ATG5-ATG7-NCOA4 pathway and IREB2 related to ferritin metabolism, and the regulatory pathways of p62-Keap1-NRF2 and HSPB1 all have effects on iron. The third category is related pathways around lipid metabolism, such as P53-SAT1-ALOX15, ACSL4, LPCAT3, etc., which have effects on lipid regulation and ferroptosis. In addition, Erastin acts on mitochondria to induce ferroptosis. Also, recent studies have shown that the FSP1-CoQ10- NAD(P)H pathway exists as an independent parallel system that works cooperatively with GPX4 and glutathione to inhibit phospholipid peroxidation and ferroptosis.

The occurrence of ferroptosis can also be regulated by sulfur transfer pathways and other pathways. Under oxidative stress, methionine can be converted into cystine through the sulfur transfer pathway, and then GSH can be synthesized to further exert its antioxidant effects^31^. In addition, the p62-Keap1-NRF2^32^, ATG5-ATG7-NCOA4^33^, and glutamine metabolic pathways^25^ can effectively regulate the formation of intracellular iron ions and ROS and play a regulatory role in ferroptosis (Fig. 2).

## Role of ferroptosis in the occurrence and development of related diseases

### Ferroptosis and tumors

#### Pancreatic cancer

Eling et al.^34^ found that artesunate (ART) specifically induced ROS production and activated ferroptosis in pancreatic ductal adenocarcinoma cell lines. In addition, the combination of Cotylenin A (CN-A) and phenylethyl isothiocyanate (PEITC) induces the production of ROS and triggers ferroptosis, thus inhibiting the proliferation of various pancreatic cancer cells (such as MIAPaCa-2 and PANC-1 cell lines). Recent studies have found that a more effective combination of Piperlongumine (PL), CN-A and sulfasalazine (a ferroptosis inducer) significantly promotes ferroptosis in the pancreatic cancer cell lines MIAPaCa-2 and PANC-1^35^.

#### Hepatocellular carcinoma (HCC)

Sorafenib is widely used in the treatment of advanced HCC, and inducing ferroptosis of HCC cells is an important mechanism for the biological effects of sorafenib. Loss of function of retinoblastoma (Rb) protein is an important event in the development of liver cancer. Louandre C et al. found that upon exposure to sorafenib, the Rb-negative status of HCC cells promotes the occurrence of ferroptosis^36^. Other studies have found that low-density lipoprotein (LDL)–docosahexaenoic acid (DHA) nanoparticles can selectively kill human HCC cells and inhibit the growth of rat HCC. After treatment with LDL-DHA nanoparticles, rat and human HCC cells underwent obvious lipid peroxidation, GSH depletion, and GPX4 inactivation before cell death, which ultimately proved to be ferroptosis^37^. In addition, Sigma 1 receptor (S1R) is abundantly expressed in hepatocytes. Inhibition of this receptor also promotes ferroptosis in HCC cells^38,39^. Furthermore, there are many negative regulators of ferroptosis in HCC, such as nuclear factor erythroid 2-related factor 2 (NRF2), metallothionein-1G (MT-1G), CDGSH iron-sulfur domain 1 (CISD1) and P53. These regulatory factors can inhibit ferroptosis in HCC cells in different ways. The p62-Keap1-NRF2 pathway plays an important role in preventing ferroptosis in HCC cells, and the RAS/Raf/MEK pathway is an important target for the treatment of HCC^32,40^. MT-1G promotes the development of sorafenib resistance by inhibiting ferroptosis, and MT-1G knockdown increases GSH depletion and lipid peroxidation^41^. CISD 1 is an iron-containing outer mitochondrial membrane protein that has protective effects on mitochondrial injury in the occurrence of ferroptosis in liver cancer cells^42^.

**Fig. 3:**
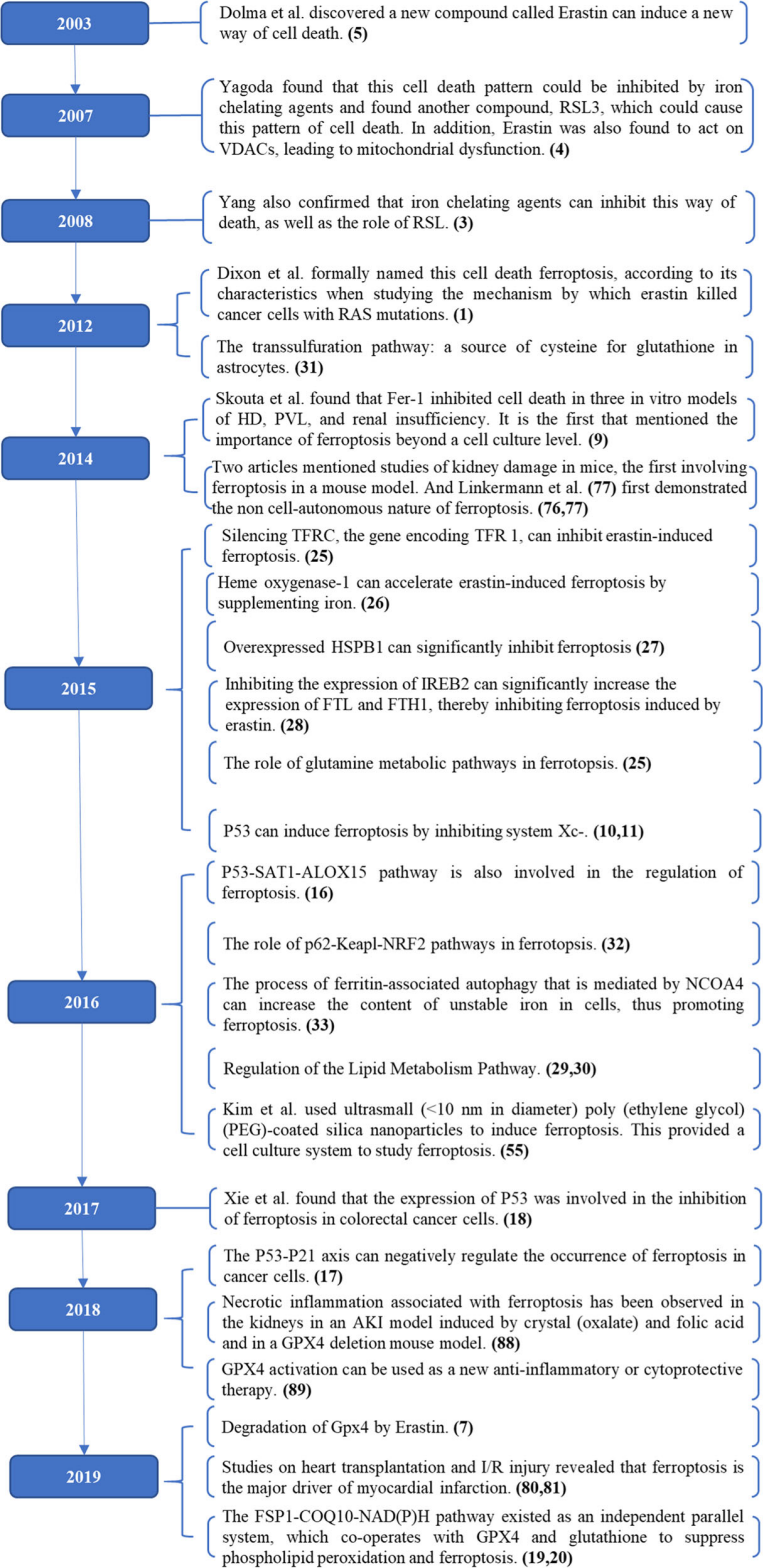
Hallmark contributions of ferroptosis.

#### Gastric cancer (GC)

Hao et al.^43^ found that erastin can induce ferroptosis in gastric GC cells and that cysteine dioxygenase type 1 (CDO1) plays a key regulatory role. CDO1 can absorb cysteine competitively, thus restricting the process of GSH synthesis and promoting ferroptosis. Inhibition of CDO1 activity restores the level of GSH in cells, prevents the production of ROS, reduces the level of lipid peroxidation, and ultimately inhibits the occurrence of ferroptosis.

#### Colorectal cancer (CRC)

Xie et al.^18^ found that P53 inhibits erastin-induced ferroptosis in CRC cells by blocking the activity of dipeptidyl-peptidase-4 (DPP4), which was different from the previous role of P53 in promoting ferroptosis in other cancer cells. P53 loss inhibits the nuclear accumulation of DPP4 and thus facilitates plasma membrane-associated DPP4-dependent lipid peroxidation, which eventually leads to ferroptosis. In addition, cisplatin has been found to induce ferroptosis and apoptosis in CRC cells, and GSH deletion and GPX inactivation are important mechanisms for this occurrence. Further studies have shown that the combination of cisplatin and the ferroptosis inducer erastin enhances the antitumor effect of drugs, indicating that ferroptosis has great potential in antitumor therapy, thus opening a new manner for the in-depth application of classical drugs^44^.

#### Breast cancer

Breast cancer is one of the leading causes of cancer-related death in women, while triple-negative breast cancer (TNBC) accounts for ~15–18% of breast cancer. TNBC currently lacks effective targeted treatment, usually based on chemotherapy, with a poor prognosis. Cystine is one of the most important amino acids in TNBC. Therefore, inhibiting the activity of system Xc- reduces cystine intake and leads to ferroptosis. However, the application of DFO and Fer-1 prevents cell death^45^. Another study found that the MUC1-C transmembrane protein is highly expressed in TNBC, which is similar to the xCT light chain of system Xc- and plays an important role in maintaining GSH level and redox balance. MUC 1-C forms a complex with xCT and the CD44 variants (CD44v), interacting with xCT to control GSH levels. Inhibition of activation of the MUC1-C/xCT signaling pathway can induce ferroptosis in TNBC cells, thereby killing tumor cells or reducing tumor cell self-renewal ability^46,47^.

#### Lung cancer

In highly differentiated lung adenocarcinomas, the iron-sulfur cluster biosynthetic enzyme NFS1 is abundantly expressed and maintains the expression level of the iron-sulfur cluster. Suppression of NFS1 alone does not induce ferroptosis, but when cells produce large amounts of ROS, iron starvation induced by inhibition of NFS1 promotes ferroptosis. Other studies have found that P53 is involved in inducing ferroptosis in lung cancer A549 cells. When erastin acts on lung cancer A549 cells, it upregulates and activates P53, thereby transcriptionally activating its downstream target genes (including P21 and bax), thereby inhibiting SLC7A11 activity, inducing ROS accumulation and eventually leading to ferroptosis^48^.

#### Clear cell renal cell carcinoma (ccRCC)

ccRCC cells are highly sensitive to the depletion of glutamine and cystine, which are required for GSH synthesis. These cells rely heavily on the GSH/GPX pathway to prevent lipid peroxidation and cell death. It has been found that inhibition of GSH synthesis in ccRCC can induce ferroptosis and inhibit the growth of tumors^49^.

#### Adrenocortical carcinomas (ACCs)

ACCs are highly malignant cancers, and mitotane is routinely used in current treatment regimens. A study found that significant increases in the expression of GPX4 and sensitivity to ferroptosis in ACCs, suggesting that inducing ferroptosis may be a promising approach for the treatment of ACCs. In addition, ferroptosis inducers may be more effective and less toxic than mitotane in treating ACC patients^50^.

#### Ovarian cancer

Exposure of ovarian cancer cells to high concentrations of ART causes ROS-dependent DNA damage and cell death, leading to G2/M phase arrest, a process often associated with ferroptosis^51^. High-grade serous ovarian cancer (HGSOC) is the most common subtype of malignant ovarian tumors. In HGSOC cells, iron metabolism is significantly interfered with, and therefore, iron uptake and retention increase, TFR1 expression of iron intake increases, iron efflux pump FPN expression is reduced and ferritin is relatively increased. The above biological processes can lead to excessive iron accumulation in the cells, which provides a basis for the occurrence of ferroptosis^52^.

#### Melanoma

In a study of melanoma, it was found that miR-137 negatively regulates ferroptosis by directly acting on the glutamine transporter SLC1A5 in melanoma cells, while knockdown of miR-137 promotes ferroptosis^53^. Other studies have found that inhibiting mitochondrial complex I triggers an increase in mitotic-dependent ROS levels, which ultimately leads to ferroptosis in melanoma cells^54^. In addition, ultrasmall (<10 nm in diameter) poly(ethylene glycol) (PEG)-coated silica nanoparticles induce ferroptosis in starving cancer cells and tumor-bearing mice^55^. This provides a new direction for the treatment of melanoma and provides a cell culture system to study ferroptosis.

#### Head and neck cancer (HNC)

In studies of HNC, two GPX 4 inhibitors, (1s, 3R)-RSL 3 and ML-162, were found to induce ferroptosis in HNC cells to varying degrees. Additionally, ART can induce ferroptosis in HNC cells, during which GSH depletion and ROS accumulation occur. Inhibition of the NRF2-ARE pathway abolishes the resistance to ART and GPX4 inhibitors, reversing the resistance of HNC cells to ferroptosis^56,57^. In addition, inhibition of the CISD2 gene in HNC cells increases the accumulation of mitochondrial iron and lipid ROS, thereby promoting the occurrence of ferroptosis^58^.

### Ferroptosis and neurological diseases

#### Neurodegenerative disorders

A large body of evidence indicates that many neurodegenerative diseases are characterized by the accumulation of local iron in specific regions of the central nervous system and/or the peripheral nervous system. This accumulation is often caused by the redistribution of cellular iron and may lead to iron-catalyzed Fenton chemistry. Studies have shown that iron accumulation and lipid peroxidation are associated with the development of various neurological diseases, accompanied by a decrease in GSH and GPX4 levels. Alzheimer’s disease (AD) is the most common neurodegenerative disorder characterized by cognitive impairment, and iron levels in the hippocampus, which are severely impaired in AD patients, are significantly elevated^59^. Abnormalities in iron homeostasis in brain tissue can induce massive production of ROS in brain cells, ultimately causing catastrophic oxidative damage to sensitive subcellular structures^60^. In mice, GPX4 knockdown leads to age-dependent neurodegenerative changes and neuronal loss, which can be exacerbated by dietary deficiency of vitamin E (a ferroptosis inhibitor)^61^. Inhibiting ferroptosis in neurons can effectively improve the prognosis of AD. The main pathophysiological feature of Parkinson’s disease (PD) is the degeneration of dopaminergic neurons in the substantia nigra pars compacta, where iron is abundant^62^. DFO alleviates oxidative stress injury and increases dopamine activity, thereby improving motor neurological symptoms and inhibiting the deterioration of motor function^63^. DFO has a certain protective effect on neurons in early PD patients. Huntington’s disease (HD) is a progressive neurodegenerative disease, and the continued accumulation of iron and abnormalities in glutamate and GSH levels are typical pathological features of HD^9,64^. The plasma GSH content in HD patients is usually low, and the GPX activity in erythrocytes decreases, which are closely related to ferroptosis^65^. The use of Fer-1 and iron chelators also has a good protective effect on neurons^9,66^. Amyotrophic lateral sclerosis (ALS) is a neurodegenerative disease affecting motor neurons in the cerebral cortex, spinal cord, and brainstem. A large amount of iron accumulation in the spinal cord can be detected in the lesion area of ALS^67^. Other studies have shown that the level of lipid peroxidation in erythrocytes of ALS patients increases and GSH level decreases, and the decrease in GSH level, in turn, exacerbates the degeneration of ALS motor neurons^68^. Friedreich’s ataxia (FRDA) is an autosomal recessive hereditary neurodegenerative disease. The amplification of GAA trinucleotides in the FRDA gene is closely related to the accumulation of iron in mitochondria. The increased levels of lipid peroxide and ROS in FRDA neurons and the decrease in GSH content increase the sensitivity to oxidants, suggesting that FRDA may be closely related to ferroptosis^69^. Periventricular leukomalacia (PVL) is associated with oligodendrocyte damage. A study found that Fer-1 inhibits the occurrence of ferroptosis by increasing the GSH level in oligodendrocytes, thus providing an effective means for the treatment of PVL^9^. These findings provide promising ideas for the treatment of neurodegenerative diseases.

#### Stroke

Ischemic stroke accounts for approximately 80% of strokes. After severe ischemic and hypoxic brain injury, iron deposition increases in the basal ganglia, thalami, periventricular, and subcortical white matter areas^70^. Studies have shown that in a mouse model of ischemic stroke, the level of GSH in neurons is significantly decreased, the degree of lipid peroxidation is enhanced, and the activity of GPX is decreased^71^. In addition, the use of ferroptosis inhibitors significantly improves the prognosis of patients with ischemic stroke^72^.

In the study of hemorrhagic stroke, N-acetylcysteine (NAC) inhibits heme-induced ferroptosis in brain cells by neutralizing toxic lipids produced by arachidonate–dependent ALOX5 activity. Early application of NAC after intracerebral hemorrhage can reduce neuronal mortality and effectively improve the prognosis of patients^73^. Other studies have found that the level of GPX4 in brain tissue decreases significantly 24 h after cerebral hemorrhage, while increasing the level of GPX4 significantly reduces neuronal dysfunction, brain edema, blood-brain barrier damage, oxidative stress and inflammatory damage after cerebral hemorrhage, and the application of the ferroptosis inhibitor Fer-1 also significantly reduces the extent of secondary brain injury after cerebral hemorrhage^74^.

#### Traumatic brain injury (TBI)

The evolution of TBI is accompanied by biological processes, such as iron accumulation, disordered iron metabolism, upregulation of ferroptosis-related genes, decreased GPX activity and ROS accumulation^75^. The application of the ferroptosis inhibitor Fer-1 significantly reduces iron deposition, neuronal degeneration, and injury and improves the prognosis of patients, thus providing an experimental basis for TBI treatment targeting ferroptosis^75^.

### Ferroptosis and acute kidney injury (AKI)

In 2014, two articles mentioned studies of kidney damage in mice, the first involving ferroptosis in a mouse model. In GPX4 knockout mice, the incidence and mortality in spontaneous AKI increased significantly^76^. In another study, Linkermann et al.^77^ found that ferroptosis was functionally relevant in vivo in acute tubular necrosis and ischemia/reperfusion (I/R) injury and showed that a ferroptosis inhibitor, a new third-generation ferrostatin (termed 16–86), protected against this injury. The study also showed that ferroptosis is independent of the necroptosis-inhibiting complex in renal tubules, specifically Fas-associated protein with death domain and caspase-8, which are important markers of spontaneous necroptosis. This study first highlights the non-cell-autonomous nature of ferroptosis. Similarly, in the nephrotoxic folic acid-induced acute kidney injury (AKI) mouse model, ferroptosis is the main death pathway of renal tubular cells, and the application of Fer-1 can effectively improve renal function^78^. In addition, HO-1 also plays an important role in inhibiting ferroptosis in renal proximal tubule cells^79^. Therefore, the therapeutic effect of AKI can be effectively improved by inhibiting the occurrence of ferroptosis.

### Ferroptosis and I/R injury

It has been found that ferroptosis inhibitors can effectively repair I/R-induced cell damage. Gao et al.^25^ found that inhibiting ferroptosis by inhibiting glutamine metabolism could be used to treat tissue damage triggered by I/R in the isolated hearts of wild-type mice. At the beginning of reperfusion, the isolated hearts were treated with DFO. Compared with heart functions in the control group, the heart function of the DFO group was significantly enhanced. Moreover, the release of lactate dehydrogenase was significantly inhibited by deferoxamine during perfusion. IR injury induced by heart transplantation is a clinically significant form of sterile inflammation. Li et al.^80^ explored the mechanism of inflammatory responses after heart transplantation and found that ferroptosis promoted the adhesion of neutrophils to coronary endothelial cells through the TLR4/Trif/type I IFN signaling pathway, thus inducing neutrophil recruitment to the injured myocardium. Moreover, Fer-1 effectively reduces the death of cardiomyocytes and blocks the neutrophil recruitment after heart transplantation. This group also used a model of coronary artery ligation-induced myocardial I/R injury, in which inhibition of ferroptosis reduced infarct size and improved cardiac function. Fang et al.^81^ demonstrated that blocking ferroptosis reduces the severity of myocardial I/R injury in cardiomyopathy. This group also studied mice with doxorubicin-induced cardiomyopathy and found that ferroptosis played an important role, possibly because doxorubicin upregulates HO-1, releasing free iron and leading to the generation of oxidized lipids in the mitochondrial membrane. Other studies have found that liproxstatin-1 can completely block lipid peroxidation and repair liver damage caused by I/R^76^.

### Ferroptosis and other diseases

Studies have shown that ferroptosis is closely related to the pathophysiological processes of more and more diseases. For example, ART treatment can significantly reduce the degree of liver injury and inhibit the formation of fibrotic scars in mice with liver fibrosis, in which the morphological characteristics of ferroptosis appear in hepatocytes^82^. Other studies have found that islets are indeed susceptible to ferroptosis in vitro. The induction of ferroptosis leads to impaired islet function, while ferroptosis inhibitors restores islet function. This provides an experimental basis for human islet transplantation and the treatment of type one diabetes^83^. In addition, ferroptosis has been found to play a regulatory role in the progression of the diseases, such as acute myeloid leukemia^84^, age-related macular degeneration (AMD)^85^, psoriasis^86^, and hemolytic disorders^87^. In addition, the relationship between ferroptosis and inflammation is also a hot research topic. Necrotic inflammation associated with ferroptosis has been observed in the kidneys in an AKI model induced by crystal (oxalate) and folic acid and in a GPX4 deletion mouse model^88^. Studies have found that GPX4 activation inhibits the activation of the arachidonic acid (AA) and nuclear factor-kappa B (NF-κ B) pathways in the lipid peroxidation-mediated inflammatory response, thereby reducing ROS levels in cells and inhibiting ferroptosis^89^. Therefore, GPX4 activation could be used as a new anti-inflammatory or cytoprotective therapy (Fig. 3).

## Issues and prospects

With the deepening of the research, ferroptosis has been found in the pathophysiological processes of more and more diseases, and it provides a new method for treating these diseases. In addition, ferroptosis, as an independent mode of cell death, can also play a role in diseases together with other types of cell death, which provides the possibility of joint application of existing treatment schemes and helps to solve drug resistance issues in some diseases. However, the study of ferroptosis is still in its infancy, and many problems remain unsolved^1^: P53 is an important regulator of apoptosis, and a large number of apoptotic factors are dependent on P53 activation to regulate the occurrence of apoptosis. P53 also plays an important role in the regulation of ferroptosis: P53 can inhibit ferroptosis induced by system Xc- by downregulating the expression of SLC7A11, and it can also inhibit ferroptosis through the P53-P21 axis under certain circumstances. Other studies have shown that autophagy also plays a role in the occurrence of ferroptosis^10,11,17^. Activation of autophagy can cause changes in ferritin. In the ATG5-ATG7-NCOA4 pathway, the process of ferritin-associated autophagy that is mediated by NCOA4 can increase the content of unstable iron in cells, thus promoting ferroptosis^33^. Therefore, there are some common points in the regulation of ferroptosis, apoptosis, autophagy, and other cell death modes. What is the relationship between these different types of cell death? Is it synergy or antagonism? Whether these various manners of cell death can be integrated into a complete regulatory network still requires further exploration^2^. Changes in iron ions play an important role in the occurrence and development of ferroptosis^1^. Free intracellular Fe^2 +^ can produce hydroxyl radicals or peroxide radicals under the action of the Fenton reaction, thus further oxidizing lipids. However, other studies have found that another important transition metal, copper, is involved in the redox metabolism of the biological system and has similar effects on glutamate-induced oxidation and erastin-mediated ferroptosis in HT22 cells. Therefore, in addition to iron, under certain conditions, other metal ions can also regulate the occurrence of ferroptosis^90,91^. This poses a serious challenge to the original concept of ferroptosis. Is iron necessary to promote the production of lipid peroxides, or can other substances take the place of iron in ferroptosis? This still requires further in-depth study^3^. Upstream iron metabolism genes (such as FPN, TFR1, and DMT1) can influence the occurrence and development of ferroptosis^25–28^, but how does the downstream pathway change? The specific molecular mechanism is still unclear^4^. Increasing evidence shows that ferroptosis can be accompanied by inflammation, and that cells can activate innate immune system factors through ferroptosis stimulation and play a role in regulating inflammation damage, signal transduction, and cell growth^88,89^. Some studies have found that GPX4 activation can be used as a new anti-inflammatory or cytoprotective therapy^89^, but how can ferroptosis promote the development of inflammation? When ferroptosis occurs, how does the downregulated GSH/GPX4 regulate the inflammatory process? These problems need to be further explored.

At present, there are still no specific markers of ferroptosis, such as there are for apoptosis (caspase activation) and autophagy (autophagy lysosome formation). Therefore, the study of specific markers of ferroptosis is of great importance. In addition to the classical pathways, are there other ferroptosis regulatory pathways? How should basic research results of ferroptosis be applied to clinical treatment? These are urgent problems to be solved.

## Conclusion

In summary, the discovery of ferroptosis has opened up a new platform in the field of disease research, and its clinical significance in the occurrence, development, and treatment of diseases has gradually emerged. At present, research on ferroptosis is still in its infancy. It is of great theoretical significance and practical value to explore the mechanism of ferroptosis and its role in various diseases and to propose effective and highly targeted therapies. This is also the future direction of ferroptosis research.
